# Antifungal and anti-biofilm activity of the first cryptic antimicrobial peptide from an archaeal protein against *Candida* spp. clinical isolates

**DOI:** 10.1038/s41598-018-35530-0

**Published:** 2018-12-04

**Authors:** Emanuela Roscetto, Patrizia Contursi, Adriana Vollaro, Salvatore Fusco, Eugenio Notomista, Maria Rosaria Catania

**Affiliations:** 1 Section of Clinical Microbiology, Department of Molecular Medicine and Medical Biotechnology, University of Naples Federico II, Via Pansini 5, 80131 Naples, Italy; 2 Department of Biology, University of Naples Federico II, Campus of Monte S. Angelo, Via Cinthia, 80126 Naples, Italy

## Abstract

*Candida* species cause cutaneous and systemic infections with a high mortality rate, especially in immunocompromised patients. The emergence of resistance to the most common antifungal drugs, also due to biofilm formation, requires the development of alternative antifungal agents. The antimicrobial peptide VLL-28, isolated from an archaeal transcription factor, shows comparable antifungal activity against 10 clinical isolates of *Candida* spp. Using a fluoresceinated derivative of this peptide, we found that VLL-28 binds to the surface of planktonic cells. This observation suggested that it could exert its antifungal activity by damaging the cell wall. In addition, analyses performed on biofilms via confocal microscopy revealed that VLL-28 is differentially active on all the strains tested, with *C*. *albicans* and *C*. *parapsilosis* being the most sensitive ones. Notably, VLL-28 is the first example of an archaeal antimicrobial peptide that is active towards *Candida* spp. Thus, this points to archaeal microorganisms as a possible reservoir of novel antifungal agents.

## Introduction

*Candida* species are the most prevalent opportunistic fungal pathogens worldwide. *Candida* spp. commonly dwell as commensal microbes colonizing the skin, oral cavities, and gastrointestinal and genital-urinary tracts of most healthy humans. When alterations in the host microbiota or in the host immune and defence system occur, *Candida* spp. can become pathogenic, causing numerous disorders ranging from cutaneous infections to severe systemic infections with a high mortality rate in hospitalized patients^1–5^.

*Candida* bloodstream infection is often associated with the presence of implanted medical devices, such as shunts, stents, prostheses, endotracheal tubes, and various types of catheters^6–8^, on which *Candida* species grow as a resilient biofilm capable of withstanding high antifungal concentrations. As expected, in patients with candidemia, biofilm-producing strains have been associated with increased morbidity and mortality compared to non-biofilm-producing ones^9^. Among *Candida* isolates, *C*. *albicans* represents the predominant specie, although, in recent years, an increasing incidence of fungal infections by non-*albicans Candida* species has been observed in hospital environments^4,10–12^. *C*. *krusei*, *C*. *tropicalis*, *C*. *parapsilosis*, *C*. *glabrata* have all been implicated in biofilm-associated infections^13,14^.

Biofilms are highly structured communities of enclosed microorganisms within a self-produced protective extracellular matrix, with biofilm-embedded cells showing properties that are distinct from planktonic cells^15–17^. In particular, the biofilm matrix acts as a protective barrier, making the microbial cells more resistant towards conventional antifungal therapeutics and the host immune system, as well as other environmental perturbations. Therefore, biofilm-forming *Candida* infections are difficult to treat, and biofilm-related (sessile) minimal inhibitory concentrations (MICs) are often extremely higher than the MICs for planktonic (non-biofilm) cells^18,19^. Furthermore, the variation of biofilm formation among *Candida* strains and/or the differential biofilm response to several antifungal classes contributes to the virulence traits. In addition to the presence of a secreted extracellular matrix, the enhanced drug resistance shown by biofilm-embedded cells is also related to (i) the local increase in cell density, (ii) the upregulation of efflux pumps, (iii) the alteration of sterols in their membranes, and iv) the activation of stress response mechanisms. This leads to the onset of persistent cells, which are a subset of metabolically dormant yeasts cells within biofilms^20–23^.

Because of the recalcitrance of *Candida* biofilms to treatment with conventional drugs, high antifungal doses in systemic therapy are needed to eradicate the infections, along with the removal of the colonized medical devices in the case of implantations^24–26^. Only three classes of antifungal drugs are currently being used to treat invasive candidiasis: azoles (i.e., fluconazole), echinocandins (i.e., caspofungin) and polyenes (i.e., amphotericin B)^27^. In recent years, the antifungal resistance of *Candida* spp., especially of non-*albicans* species, has been observed. For example, *C*. *krusei* was found to have a reduced susceptibility to fluconazole, while *C*. *glabrata* was reported to be resistant to both azoles and echinocandins^28^. Since amphotericin B resistance in *Candida* species remains extremely rare, this drug is the treatment of choice in monotherapy for life-threatening systemic candidiasis. However, with the onset of antifungal resistant pathogens, there is an increasing need to design new antimycotics and/or discover alternative agents that improve the fungicidal activity of the current antifungals.

Antimicrobial peptides (AMPs) with their extraordinary properties, such as broad-spectrum activity, rapid action and unlikely development of resistance, have become promising molecules as new antibiotics^29,30^. An example of such molecules is represented by cationic antimicrobial peptides (CAMPs), i.e., short and positively charged peptides with an amphipathic structure. CAMPs are active against Gram-positive and Gram-negative bacteria, as well as fungi and protozoa^29–31^. Regardless of their specific mechanism of action, the interaction of CAMPs with the bacterial cell membrane is the key step, which eventually leads either to the disruption of the membrane integrity or to the alteration of its electrochemical potential^31,32^. Interestingly, some CAMPs also exhibit toxicity towards eukaryotes, such as fungi, despite the different lipidic membrane compositions and the distinct structures of the cell wall^33^.

While the effect of some AMPs on biofilm formation, as well as their antimicrobial activity on biofilm-embedded cells, has already been investigated^34–36^, only a limited number of studies describes the effect of CAMPs on biofilm formation by fungal pathogens such as *Candida*^37,38^. Recently, we identified a cryptic CAMP-like peptide (designated as VLL-28) in the sequence of the archaeal transcription factor Stf76^39^, which is encoded by the hybrid plasmid–virus pSSVx infecting *Sulfolobus islandicus*^40–42^. The peptide was identified using an *in silico* tool developed to search for cryptic antimicrobial peptide-like sequences hidden in the primary structure of proteins^32,43–46^. VLL-28 displays chemical, physical and functional properties typical of CAMPs and acquires a defined structure in the presence of membrane mimetics^47,48^. Notably, this CAMP turned out to be toxic not only to Gram-negative and Gram-positive bacteria but also to *C*. *albicans*^47^. Therefore, in this study, we investigated the antifungal activity of VLL-28 towards pathogenic *C*. *albicans* and non-*albicans Candida* spp. isolated from blood infections. In particular, we show the *in vitro* ability of VLL-28 to (1) inhibit yeast cells growth in a planktonic state, (2) prevent cell adhesion, and (3) eradicate established biofilms.

## Results

### Antifungal activity of VLL-28 towards *Candida* spp. planktonic cells

MIC values for amphotericin B, anidulafungin, micafungin, caspofungin, 5-fluorocytosine, posaconazole, voriconazole, itraconazole, and fluconazole of 10 clinical isolates of *Candida* spp., as well as the reference strain *C*. *albicans* ATCC10231, are reported in Table S1. The antifungal activity of VLL-28, expressed as MIC and MFC values against the same panel of isolates, is shown in Table 1. Among them, the planktonic cells of *C*. *tropicalis* were the most susceptible to the peptide VLL-28 (MIC = 12.5 µM), followed by *C*. *albicans*, *C*. *parapsilosis* and *C*. *krusei* (MIC = 25 µM), while *C*. *glabrata* planktonic cells were the least sensitive, showing the highest MIC value of 50 µM. In addition, the activity of VLL-28 was also investigated in terms of minimum fungicidal concentration (MFC). The values were two-fold higher than the MIC values for all *Candida* species, except *C*. *albicans 80* and *C*. *tropicalis* isolates for which the MFC values were four-fold higher than the corresponding MICs (Table 1).

**Table 1:** Minimal fungicidal concentration (MFC) and minimal inhibition concentration (MIC) of amphotericin B and VLL-28 against *Candida* species.

Yeasts	amphotericin B		VLL-28	
	MIC μg/mL	MFC μg/mL	MIC μg/mL (μM)	MFC μg/mL (μM)
*C*. *albicans ATCC10231*	0.25	1	88.5 (25)	177 (50)
*C*. *albicans 80*	0.5	2	88.5 (25)	354 (100)
*C*. *albicans 81*	0.25	2	88.5 (25)	177 (50)
*C*. *parapsilosis 3*	0.5	2	88.5 (25)	177 (50)
*C*. *parapsilosis 10*	0.5	2	88.5 (25)	177 (50)
*C*. *tropicalis 54*	0.5	4	44.25 (12.5)	177 (50)
*C*. *tropicalis 2*	0.5	4	44.25 (12.5)	177 (50)
*C*. *glabrata 28*	0.25	2	177 (50)	354 (100)
*C*. *glabrata 34*	0.25	1	177 (50)	354 (100)
*C*. *krusei 1*	0.5	2	88.5 (25)	177 (50)
*C*. *krusei 14*	1	2	88.5 (25)	177 (50)

### *In vitro* biofilm formation assay

**Figure 1:**
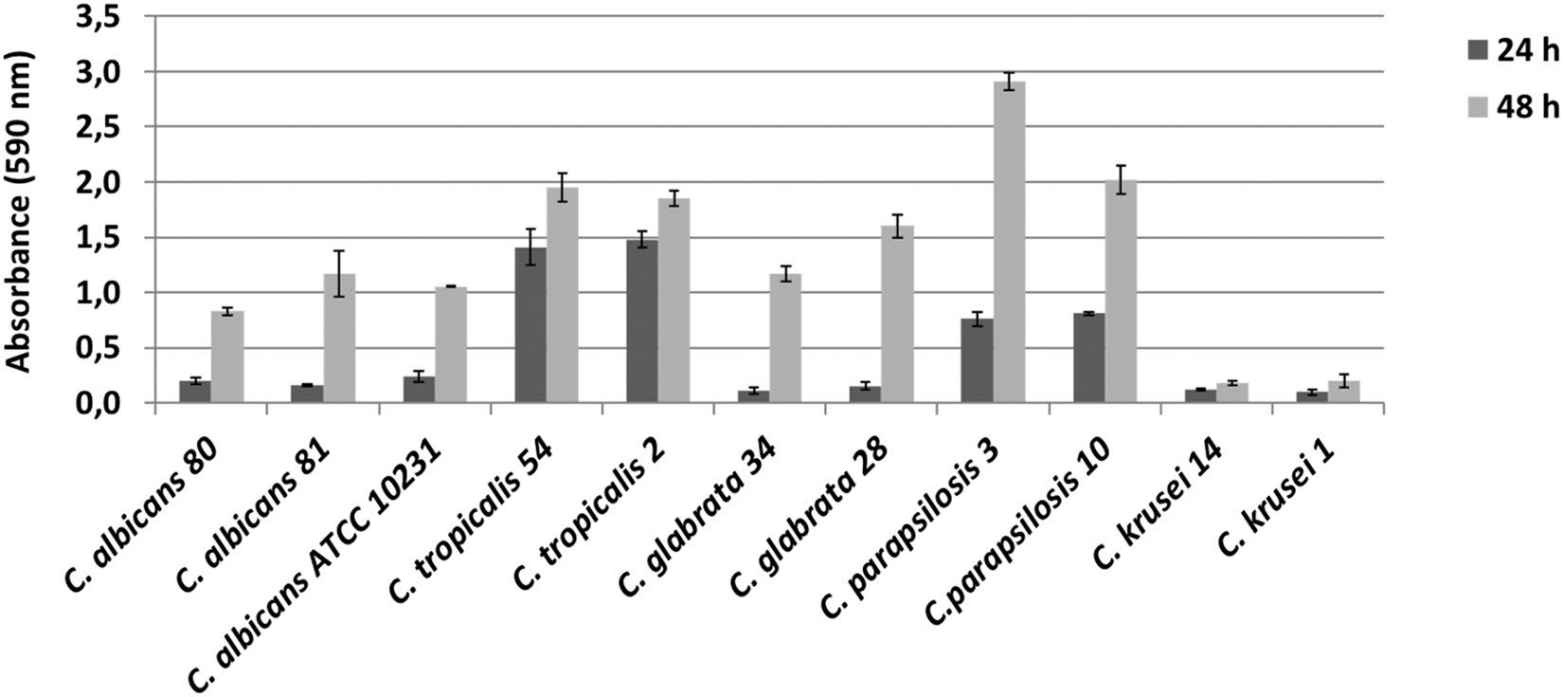
*In vitro* biofilm production. *Candida* isolates were grown in 96-well microtitre plates and their biofilm was quantified through staining with crystal violet after 24 and 48 hours of cultivation. Each value is the average of three independent experiments in triplicate. Error bars are the standard deviations.

*Candida* species were tested for the ability to produce biofilm using the crystal violet staining method, and the results are shown in Fig. 1. All the tested isolates turned out to be biofilm producers. At 24 hours, *C*. *glabrata* 34, *C*. *krusei* 14 and 1 were weak producers, all *C*. *albicans* isolates and *C*. *glabrata* 28 were moderate producers, and all *C*. *tropicalis* and *C*. *parapsilosis* were strong producers. At 48 hours, all *Candida* species, with the exception of the *C*. *krusei*, were strong producers.

### Cellular localization of VLL-28

**Figure 2:**
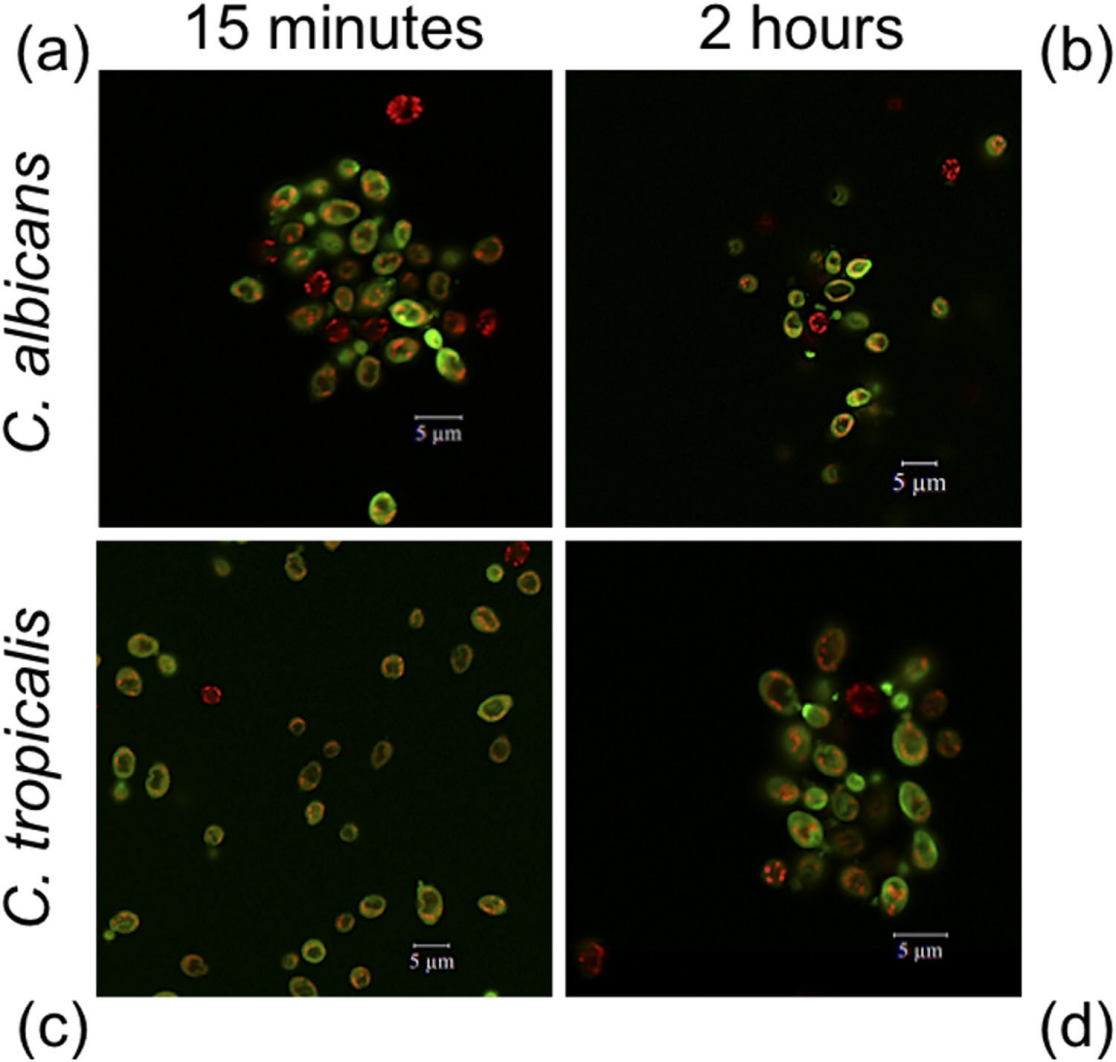
VLL-28 intracellular localization detected using confocal laser scanner microscopy. *Candida* cells were incubated in PBS at different times with a dose of the peptide corresponding to their MIC values. VLL-28 conjugated with FITC is coloured in green, while mitotracker is in red. Images of *C*. *albicans* and *C*. *tropicalis* cells are shown at 15 min (**a**,**c**) and 2 hours (**b**,**d**) posttreatment.

A fluorescein-labelled derivative of VLL-28 (VLL-28*) was used to study the cellular localization of the peptide in *C*. *albicans* and *C*. *tropicalis* using confocal laser scanning microscope (CLSM)^49^. *C*. *albicans* and *C*. *tropicalis* were chosen because these two strains are among the most sensitive to VLL-28 activity. The effectiveness of VLL-28^*^ was found to be similar to that of the not labelled peptide^50^. *C*. *albicans* and *C*. *tropicalis*, pre-incubated with MitoTracker Orange, were treated with VLL-28^*^ at the concentration of 12.5 μM and 25 μM, respectively, for 15 min and 2 h. Confocal images showed that, for both strains, the green signal (VLL-28^*^) was uniformly localized at the cellular surface of the treated cells after 15 min and did not overlap with the red (MitoTracker) signal (Fig. 2). This indicates that the peptide interacts with the fungal surfaces, probably binding to the negatively charged phospholipids, and that no internalization occurs.

### Inhibition of biofilm formation by VLL-28

**Figure 3:**
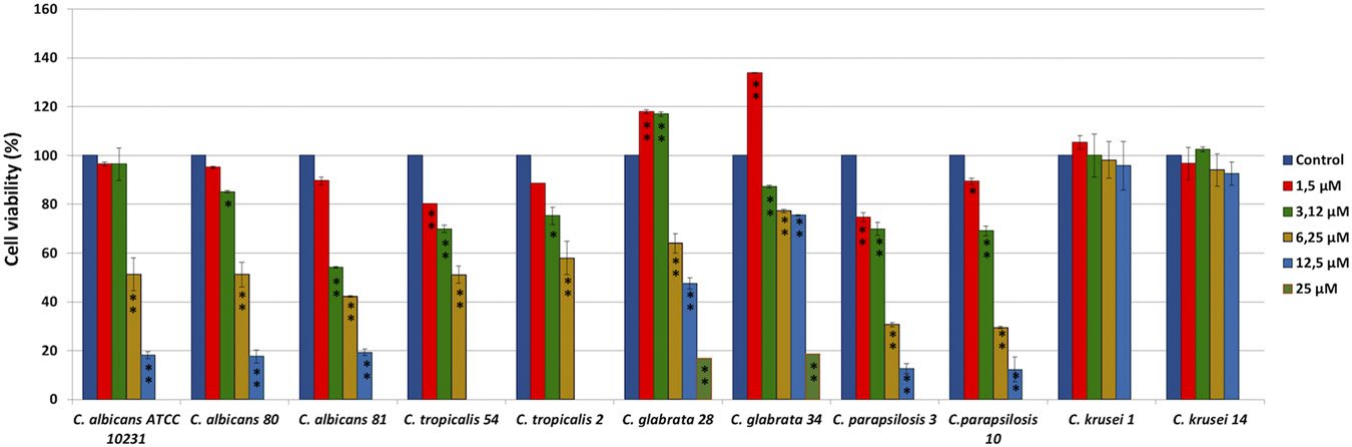
Effect of different concentrations of VLL-28 on cell viability of *Candida* species. Cell viability within the biofilm was assessed by measuring the reduction of XTT. The results are reported as the percentage relative to the untreated control. Technical and biological triplicates were conducted for all experiments, and statistical significance was determined using one-way ANOVA and Dunn’s test. *P value between 0.01 and 0.001; **P value < 0.001.

Cell adhesion either on the host cell tissues or on abiotic surfaces is the first step during *Candida* biofilm formation. Therefore, we investigated whether VLL-28 could prevent biofilm production by interfering with the cell viability at sub-MIC concentrations. The minimum biofilm inhibitory concentration (MBIC) was determined by quantifying the metabolic activity of the adherent cells using the XTT assay (Fig. 3). The percentage of biofilm viability was strongly reduced for *C. tropicalis,* *C*. *albicans* and *C*. *parapsilosis* strains, with MBIC values of 12.5 µM, and for *C*. *glabrata* strains, with MBIC values of 25 µM, while no inhibition of cell adhesion was observed for both *C*. *krusei* isolates (Fig. 3).

### Activity against preformed biofilms of *Candida* species

**Figure 4:**
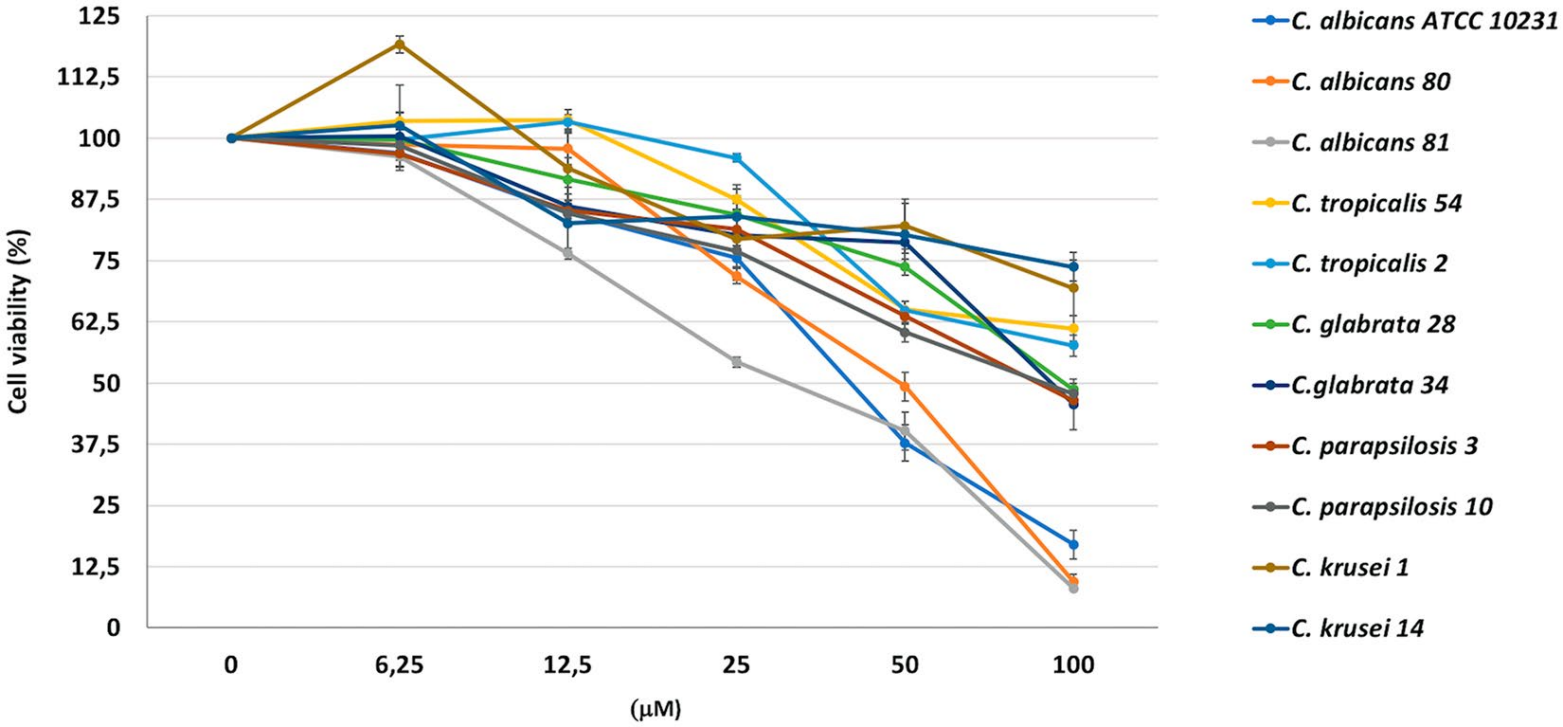
Antifungal activity of VLL-28 on cells in preformed *Candida* biofilms. Mature biofilms were exposed to the peptide at concentrations ranging from 6.25 to 100 μM for 24 h. Each data point shows the XTT activity of the VVL-28-treated biofilm normalized to the control (untreated), which was considered to be 100%. Technical and biological triplicates were run for all experiments. Statistical significance was determined using one-way ANOVA and Dunn’s test (for P values see Table S3).

Biofilm formation in *Candida* confers to the embedded cells an increased resistance towards antifungal agents. Therefore, we extended our investigation to the anti-biofilm activity of VLL-28 on preformed *Candida* biofilms. One-day-old biofilms were exposed to increasing doses of the peptide, and its antifungal activity was quantified using the XTT reduction assay. VLL-28 effectively reduced the viability of the cells embedded in the mature biofilms, with MBEC_50_ and MBEC_80_ of 50 μM and 100 μM, respectively, for *C*. *albicans* isolates and the reference strain. In addition, VLL-28 killed 50% of the cells in the preformed biofilm (MBEC_50_) of *C*. *glabrata* and *C*. *parapsilosis* isolates at a concentration of 100 μM. On the other hand, VLL-28 was less effective against the mature biofilms of *C*. *tropicalis* and *C*. *krusei* isolates (Fig. 4, Table S3).

**Figure 5:**
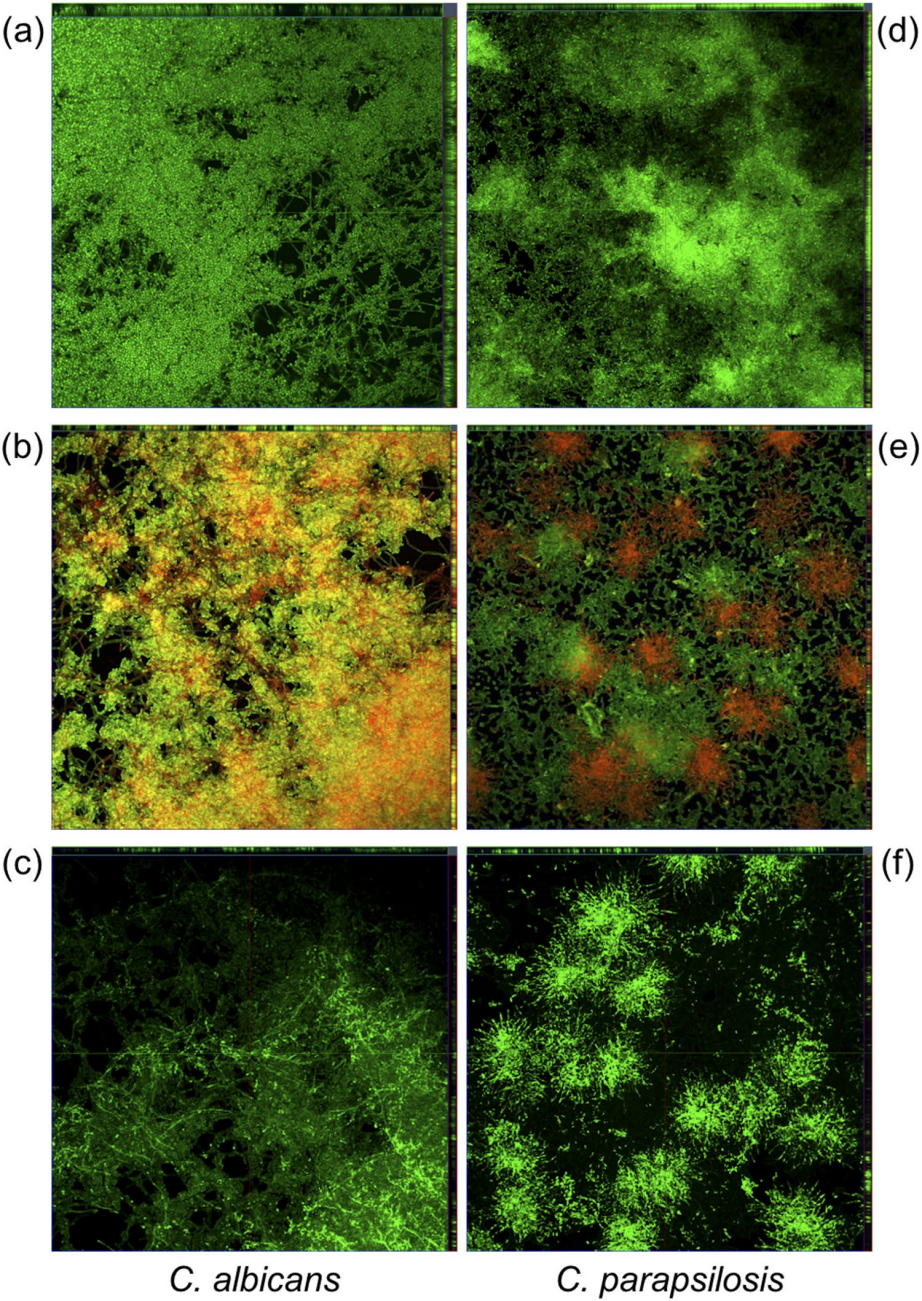
CLSM of VLL-28 on preformed biofilm of *C*. *albicans* (**a**–**c**) and *C*. *parapsilosis* (**d**–**f**). Panels a and d show untreated biofilms stained using the LIVE/DEAD FungaLight Yeast Viability Kit. Panels b and e show biofilms treated with VLL-28 (50 µM) and stained as in a and d. Panels c and f show biofilms treated with VLL-28*.

**Figure 6:**
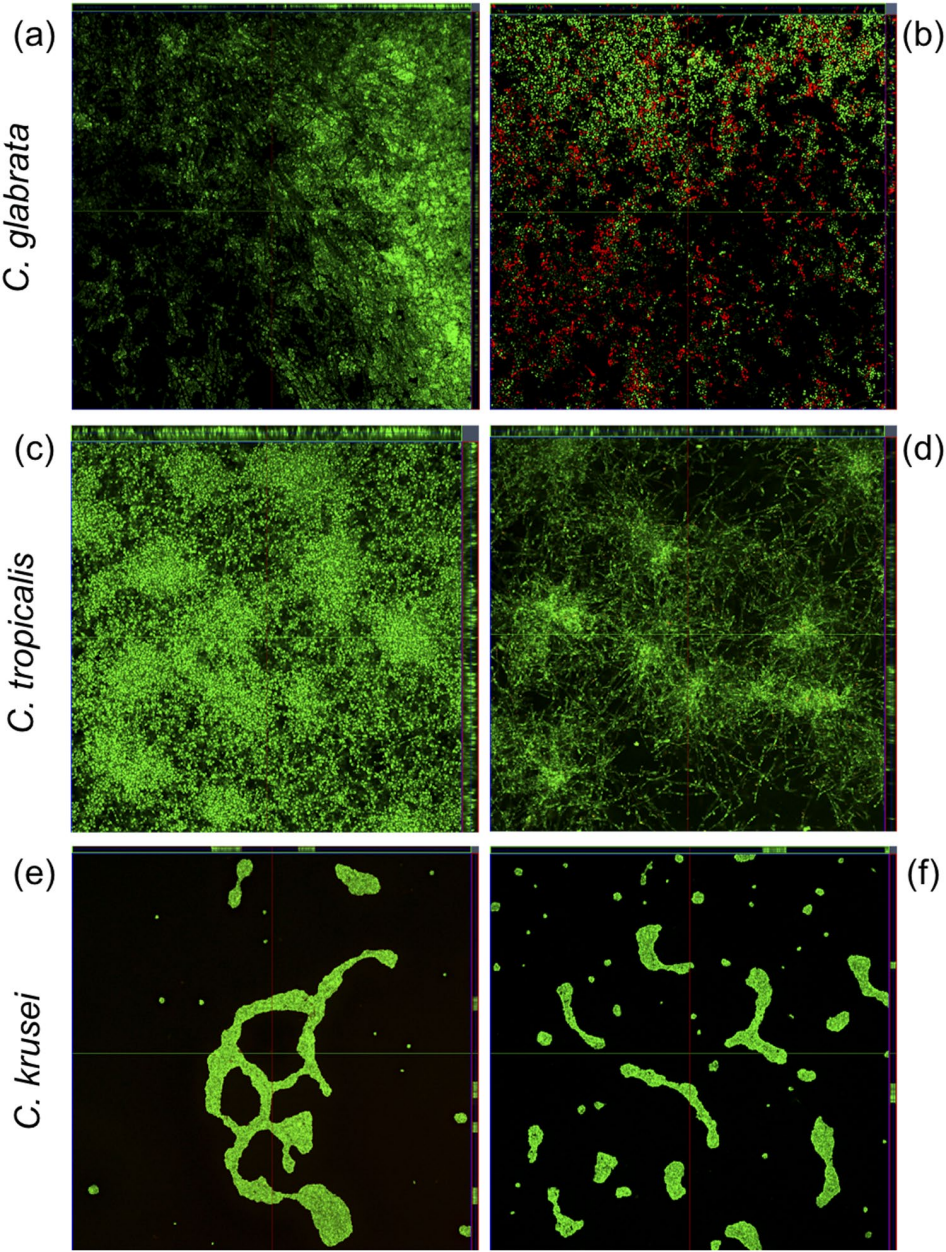
CLSM of VLL-28 on preformed biofilm of *C*. *glabrata* (**a**,**b**), *C*. *tropicalis* (**c**,**d**) and *C*. *krusei* (**e**,**f**). Panels a, c and e show untreated biofilms. Panels b, d and f show biofilms treated with VLL-28 (50 µM). All the biofilms were stained using the LIVE/DEAD FungaLight Yeast Viability Kit.

CLSM was used to investigate the effect of VLL-28 on *Candida* preformed biofilms. Biofilms were grown on Nunc® Lab-Tek® II chambered cover glasses and stained using the LIVE/DEAD FungaLight Yeast Viability Kit. As shown by the differential staining with SYTO9 (green fluorescence, live cells) and propidium iodide (red or yellow-red fluorescence, dead cells), VLL-28 caused the death of most of the cells embedded in the mature biofilms of *C*. *albicans* (Figs 5a,b and S1a,b) and decreased the cell viability in those of *C*. *parapsilosis* (Figs 5d,e and S1d,e) and *C*. *glabrata* (Fig. 6a,b and S2a,b). In the case of *C*. *tropicalis* biofilm, images showed a thickness reduction of approximately 30% and the appearance of regions with lower density (Figs 6c,d and S2c,d) compared to the untreated control. However, viable cells were detectable, which suggests that the peptide could have a fungistatic effect on the preformed biofilm of *C*. *tropicalis*. Regarding *C*. *krusei*, VLL-28 administration did not cause any visible change in the thicknesses and viability of its mature biofilm, which is consistent with what was observed both by CLSM imaging and by the XTT reduction assay (Figs 6e,f and S2e,f).

To verify whether the different effects on *Candida* biofilms could be traced back to the different ability of VLL-28 to penetrate the biofilm matrix, *C*. *albicans* and *C*. *parapsilosis* biofilms were treated with VLL-28* and analysed using CLSM. As shown, the distribution of VLL-28* mirrors that of the dead cells (red spots), i.e., the propidium iodide stained areas (Figs 5c,f and S1c,f).

## Discussion

The high risk of invasive fungal infections within the increasing population of immunocompromised patients, along with the emergence of resistance to the most common antifungal drugs in *Candida* spp., also due to biofilm phenotypes, requires the development of new antifungal agents. Antimicrobial peptides might represent a very promising alternative, not only to treat planktonic *Candida* but also for biofilm-embedded *Candida* cells^51^.

Most of the currently used antifungal agents show a specific mechanism of action. For instance, azoles act by interfering with the biosynthesis of membrane ergosterol^52,53^, while echinocandins block the synthesis of cell wall 1,3 beta-glucan, and polyenes bind ergosterol and disrupt membrane integrity^53,54^. In contrast, amphipathic AMPs exert their antimicrobial activity by binding the cellular membrane and then increasing its permeability^31,32^. Unlike classic antifungal drugs, the peculiar mechanism of action of AMPs hinders the development of microbial resistance.

VLL-28 is a cryptic antimicrobial peptide derived from a transcriptional factor of *Sulfolobus islandicus*^39^. A previous study has shown the ability of VLL-28 to inhibit the growth of bacteria and fungi in planktonic form^47^. In this study, we investigated the antifungal and anti-biofilm activity of VLL-28 against clinical isolates of *C*. *albicans* and non-*albicans* species. VLL-28 exhibited fungicidal activity against almost all the planktonic *Candida* spp. tested, with MIC values in the range of 12.5–50 µM. These data are comparable to the MIC values reported for several other natural and artificial AMPs^55–60^, as reported in Table S2. VLL-28 displayed a reduced activity only towards *C*. *glabrata*, which showed the highest MIC and MFC values (Table 1). The poor susceptibility of *C*. *glabrata* to various CAMPs has already been reported^61^ and may reflect the unique and distinctive features of the cell wall of this pathogen^62^. Interestingly, VLL-28 also exhibited strong fungicidal activity towards the planktonic cells of *C*. *krusei*, which is regarded as a potentially multidrug-resistant (MDR) pathogen. This is a naturally resistant specie to fluconazole (FLC), which has shown an increased resistance to both flucytosine and amphotericin B^63^, as well as cross-resistance to azoles^64^. In addition, the clinical failure of caspofungin towards *C*. *krusei* infections has been reported in recent years^65^.

The results obtained with the fluorescein-labelled peptide indicate that VLL-28 interacts primarily with the fungal surfaces. In fact, no internalization was observed. This confirms that VLL-28 behaves as a typical CAMP by damaging the cell membrane and/or the cell wall, thus, greatly decreasing the likelihood of the appearance of strains resistance.

The ability of *Candida* spp. to adhere to epithelial or endothelial surfaces, as well as to implanted medical devices by forming resilient biofilms, is an important virulence trait that promotes the persistence of the infection. Compared with planktonic cells, *Candida* biofilms are characterized by increased resistance to conventional antifungal drugs, in particular to amphotericin B and fluconazole^66–68^. Several factors have been suggested to be involved in the resistance of biofilm to antimicrobial drugs, including the expression of conventional resistance genes, such as those coding for efflux pumps^69^. In addition, the production of an extracellular matrix, which is a specific mechanism of the biofilm growth mode, limits drug penetration or even binds the antifungal agent and prevents it from reaching its cellular target^20,21,70^. Thus, an extremely limited drug arsenal is available to treat biofilm-related *Candida* infections. VLL-28 effectively prevents biofilm formation by reducing the cells’ adhesion to the abiotic surfaces of all the strains tested (except for *C*. *krusei*) at a concentration of the peptide that is 50% of the MIC value. The ability of this peptide to prevent biofilm formation is particularly important for medical device implantations. In fact, the microbial colonization of indwelling medical implants with subsequent biofilm formation can lead to severe complications associated with increased morbidity and mortality, such as bloodstream infections and systemic inflammation^8^.

Infection by non-*albicans Candida* species is currently a highly active research area, as these species are becoming increasingly prevalent^69^. Notably, VLL-28 reduces the metabolic activity, not only of mature biofilms formed by *C*. *albicans* (80%) but also of those formed by *C*. *glabrata* and *C*. *parapsilosis* (50%), at concentrations from 2- to 4-fold higher than those needed for the planktonic state. This result is not unexpected because even conventional antifungals that exhibit anti-biofilm activity have MBECs several-fold higher than the MICs for the same isolates^71–73^. Furthermore, our finding is similar to those reported for several other AMPs (Table S2).

CLSM images showed a diffuse cell permeabilization in 1-day-old biofilm of *C*. *albicans* and *C*. *glabrata* treated with sub-MBEC values of VLL-28, while the effect on the mature biofilm cells of *C*. *parapsilosis* was focal. The fluorescein-labelled peptide distribution appeared to overlap permeabilized cell zones. The diffuse or focal VLL-28 toxicity may depend on the different distributions of the cells on the abiotic surfaces after 24 hours of biofilm formation. In addition, VLL-28 caused a significant decrease of the biofilm biomass of *C*. *tropicalis*, but no cell killing was observed in this case. This result may be explained by the ability of VLL-28 to affect the matrix structure/stability by interacting with one or more components, thus inducing disaggregation of the biofilm without cell killing. It is worth noting that drug diffusion to the cell community within a given biofilm can be affected not only by the overall extent of the matrix itself but also by its chemical nature^74^. Indeed, Al-Fattani and Douglas^74^ demonstrated that *C*. *tropicalis* strains could form a compact extracellular matrix containing hexosamine-rich polysaccharides, which is poorly penetrated by antifungal agents.

In conclusion, we showed the ability of VLL-28 to exert antifungal activity against planktonic cells and mature biofilm of clinical isolates of *C*. *albicans* and non-*albicans* species, including *Candida krusei*, which is intrinsically resistant to fluconazole^75^. These results appear relevant and deserve further study with the perspective of developing alternative and/or complementary antifungal therapies.

## Materials and Methods

### Yeast strains and culture conditions

The *Candida* species evaluated in this study included the *C*. *albicans* ATCC 10231 reference strain and eight clinical isolates (Table 1) belonging to a collection of fungal strains previously established at the Department of Molecular Medicine and Medical Biotechnology (University of Naples Federico II). Identification was performed by subcultures on chromogenic agar (Chromid agar) (Becton Dickinson) and by biochemical characterisation using the Vitek II system (Biomerieux) and was confirmed by MS MALDI-TOF (Bruker).

Susceptibility to amphotericin B, anidulafungin, micafungin, caspofungin, 5-fluorocytosine, posaconazole, voriconazole, itraconazole, and fluconazole was assessed using the Sensititre Yeast One colorimetric microdilution method (Thermofisher). All strains were stored as 15% (v/v) glycerol stocks at −80 °C. Prior to each experiment, cells were subcultured from the stocks onto Sabouraud dextrose agar (SDA) (Becton Dickinson) at 37 °C for 48 h.

### Peptides

The peptide VLL-28 (VLLVTLTRLHQRGVIYRKWRHFSGRKYR) and its fluoresceinated derived form (VLLVTLTRLHQRGVIYRKWRHFSGRKYRGK*) (VLL-28*), bearing the chromophore fluorescein coupled to the last lysine residue, were synthetized and purified to 95% homogeneity by Inbios (Napoli, Italy), as confirmed by LC–MS analysis.

### Determination of the minimum inhibitory concentration (MIC) and the minimum fungicidal concentration (MFC)

The antifungal activity of VLL-28 was determined using a standardized broth microdilution method (Clinical and Laboratory Standards Institute (CLSI) document M27-A2)^76^. Briefly, for each *Candida* species, the cell suspension was adjusted to 3 × 10^3^ CFU/mL using a morpholinepropanesulfonic acid (MOPS)-buffered RPMI 1640 medium (R6504 - Sigma) supplemented with 0.2% (w/v) glucose. One hundred microlitre aliquots of these cell suspensions were dispensed into 96-well microtitre plates. Peptide stock solution was serially diluted using the same RPMI 1640 medium and added to the wells at a final concentration ranging from 3 µM to 100 µM, and the plate was incubated for 48 hours at 37 °C. Absorbance at 595 nm was measured using a microplate reader (Biorad mod 680). Amphotericin B at concentrations ranging from 0.25 to 2 µg/mL was chosen as the positive control because all the strains were sensitive to this agent according to the Sensititre Yeast One test.

The MIC was defined as the lowest concentration of the peptide that resulted in 90% growth inhibition after 48 h of incubation. The test was conducted at least three times using independent cell suspensions. The minimum fungicidal concentration (MFC) was determined by transferring 50 µl aliquots of each sample, previously treated with concentrations equal to or higher than the MIC, onto SDA plates and incubating the plates at 37 °C for 24 h. The lowest peptide concentraiton that yielded no fungal growth on agar plates was defined as the MFC.

### *In vitro* biofilm formation assay

Biofilms of *Candida* spp. were formed in flat-bottomed 96-well microplates as described by Stepanovic with some modifications^77^. For each strain, a cell suspension in RPMI 1640 medium supplemented with 2% (w/v) glucose was adjusted to 1 × 10^6^–5 × 10^6^ CFU/mL as determined by cell counts using a haemocytometer Neubauer improved chamber. Plate wells were inoculated with 200 µL of standardized yeast suspension in triplicate and incubated at 37 °C for 90 minutes to allow cell adhesion. A negative control was prepared by inoculating 200 µL of a yeast suspension inactivated by boiling. After the adhesion phase, non-adherent cells were removed by thoroughly washing the wells with 0.15 M sterile phosphate-buffered saline (PBS, pH 7.2). Each well was then filled with 200 µL of fresh RPMI 1640, and the plate was incubated at 37 °C for 24 h to allow biofilm formation.

To assess biofilm formation, the culture broth was gently aspirated, and each well was washed twice with PBS and dried at 60 °C for 30 minutes. The biofilm was stained by incubation for 30 min with 50 µL of a 1% (w/v) crystal violet solution. Any excess of crystal violet was removed by washing with PBS before adding 150 µL of absolute ethanol to release the dye from the biofilm. The absorbance was measured at 590 nm using a Biophotometer (Eppendorf) and was related to the amount of biofilm produced. We used the classification introduced by Stepanović *et al*.^77^ with some modifications. The isolates tested were classified into four categories: non-adherent (NA), weakly adherent (WA), moderately adherent (MA), or strongly adherent (SA).

### 2,3-bis(2-methoxy-4-nitro-5-sulfo-phenyl)-2H-tetrazolium-5-carboxanilide (XTT)-reduction assay

The (XTT)-reduction assay has been used as a routine tool for the quantitative measurement of bacterial and fungal metabolic activity, growth and response to antimicrobial treatments^78–82^. After peptide treatment, the medium was aspirated from each well to remove floating cells, and the wells were thoroughly washed twice with PBS. The assay was conducted as described by Barra *et al*.^80^ with some modifications. Two hundred microlitres of XTT solution was added to each well, and the plate was incubated in the dark for 30 min at 37 °C. Changes in the absorbance of XTT were measured spectrophotometrically at 490 nm using a microtitre plate reader (Biorad). An XTT cell proliferation Kit II was purchased from Roche Diagnostics. Viability ratios were computed for each well with respect to their relative controls.

### Confocal laser scanning microscopy

CLSM was used to illustrate the effect of peptide (50 µM) on the viability and architecture of mature (24 h) biofilms of *Candida* species. Biofilm-forming *Candida* cells were grown on Nunc® Lab-Tek® II chambered cover glasses (Sigma), and the antifungal biofilm susceptibility was assayed as described above.

Biofilms were stained with two nucleic acid dyes using the LIVE/DEAD FungaLight Yeast Viability Kit: SYTO 9 and propidium iodide (PI) (Molecular Probes). SYTO 9 penetrates both viable and nonviable cells, while PI penetrates only cells with damaged membranes (i.e., nonviable cells) and quenches the fluorescence emitted by SYTO 9. Dead and viable cells emit yellow-red and green fluorescence, respectively. Images were captured using an LSM 710 inverted confocal laser-scanning microscope (Zeiss) and analysed using CLSM Z-Stack analysis: depth measurements were taken at regular intervals across the biofilm, and three-dimensional images of mature biofilms were captured.

### Cellular localization studies of VLL-28

Confocal laser-scanning fluorescence microscopy was used to study the intracellular target of the peptides^49^. Double staining of the *C*. *albicans* and *C*. *tropicalis* strains with FITC-labelled peptides and MitoTracker Orange (chloromethyl-H_2_-tetramethyl rosamine, Molecular Probes), a permanent mitochondrion-selective dye, was achieved as follows: a *Candida* cell suspension (200 µL; 3.2 × 10^6^ cells/mL of PBS) was incubated with 150 nM MitoTracker Orange for 15 min at 37 °C. The cells were washed with 200 µL of PBS and treated for 15 min and 2 h with 25 µM and 12.5 µM FITC-labelled peptides for *C*. *albicans* and *C*. *tropicalis*, respectively. The cells were collected using centrifugation (5 min at 10,000 × *g*), suspended in 20 µL of PBS and examined by confocal microscopy using an LSM 710 confocal laser-scanning microscope equipped with a 63X objective lens.

### Adhesion inhibition assay

The adhesion of *Candida* spp. was assayed using flat-bottomed 96-well microplates. For each isolate, 100 µL of cell suspension in RPMI medium adjusted to 1 × 10^6^ CFU/mL was incubated with 100 µL of RPMI containing serially double-diluted peptide concentrations in order to obtain the final sub-MIC concentrations for each yeast, ranging from 1.5 µM to 25 µM. The plate was then incubated at 37 °C with a shaking rate of 100 rpm; the positive control consisted of peptide-free wells. After a 60 min adhesion phase, the medium with unbound peptide was aspirated; non-adherent cells were removed by washing the wells with PBS, and 200 µL of fresh RPMI was added. The plate was incubated further at 37 °C for 24 h, and an XTT reduction assay was performed as described below.

The adhesion inhibitory activity of the peptide is referred to as the minimum biofilm inhibitory concentration (MBIC), which is defined as the minimum peptide concentration leading to an 80% reduction of biofilm formation compared to a peptide-free control sample.

### Antifungal susceptibility testing of 24 h-old *Candida* biofilms

*Candida* biofilms were produced as described above; upon mature biofilm formation, the medium was aspirated, and each well was washed twice gently with 200 µL of PBS to remove planktonic cells. Peptide aliquots (200 µL per well) ranging from 6.25 to 100 µM were added, and the plate was incubated for 24 h. Peptide-free wells were included as positive controls. The biofilm formation at 24 h and 48 h was quantified using the XTT reduction assay described below. The anti-biofilm activity of the peptide is referred to as the minimum biofilm eradication concentration (MBEC), which is defined as the minimum peptide concentration resulting in 80% disruption of the biofilm compared to a peptide-free control culture.

### Statistical analysis

All experiments were performed in triplicate with the average and standard deviation calculated for all measurements. Statistical differences among the groups of data were analysed by one-way ANOVA using Prism (version 7.00 for Windows; GraphPad Software, San Diego, CA) and Dunn’s test. In all the comparisons, a P value of 0.05 or lower was considered significant.

## Electronic supplementary material

Supplementary Information

## Data Availability

The datasets used and/or analysed during the current study are available from the corresponding authors on reasonable request.
