# Supplementary material for: Antifungal and anti-biofilm activity of the first cryptic antimicrobial peptide from an archaeal protein against Candida spp. clinical isolates
Source: Sci Rep. 2018 Dec 4;8:17570. doi: 10.1038/s41598-018-35530-0 (PMC6279838; doi:10.1038/s41598-018-35530-0)

**Supplementary information to:**

**“Antifungal and anti-biofilm activity of the first cryptic antimicrobial peptide from an archaeal protein against *Candida* spp. clinical isolates”**

**Emanuela Roscetto^1^, Patrizia Contursi^2#^, Adriana Vollaro^1^, Salvatore Fusco^2^, Eugenio Notomista^2^*, and Maria Rosaria Catania^1^***

**^1^**Section of Clinical Microbiology, Department of Molecular Medicine and Medical Biotechnology, University of Naples Federico II, Via Pansini 5, 80131 Naples, Italy

^2^Department of Biology, University of Naples Federico II, Campus of Monte S. Angelo, Via Cinthia, 80126 Naples, Italy.

***These authors equally contributed to the work**

**^#^Correspondence: contursi**@**unina.it**

**Table S1. MIC values (µg/mL) of several antifungal agents against the *Candida*** species tested in this study.

|  | **Antifungal agent*^a^*** | | | | | | | | |
| --- | --- | --- | --- | --- | --- | --- | --- | --- | --- |
|  | **AND** | **MF** | **CAS** | **FC** | **PZ** | **VOR** | **IZ** | **FZ** | **AB** |
| *C. glabrata* 34 | 0.03 | 0.015 | 0.12 | <0.06 | 0.5 | 0.25 | 0.5 | 8 | 0.25 |
| *C. glabrata* 28 | 0.06 | 0.03 | 0.12 | 0.12 | 0.5 | 0.25 | 0.12 | 8 | 0.25 |
| *C. krusei* 14 | 0.03 | 0.125 | 0.12 | 8 | 0.25 | 0.5 | 0.25 | 256 | 1 |
| *C. krusei* 1 | 0.06 | 0.125 | 0.06 | 8 | 0.25 | 0.5 | 0.25 | 256 | 0.5 |
| *C. parapsilosis* 3 | 2 | 2 | 0.25 | <0.06 | 0.015 | <0.008 | 0.03 | 0.25 | 0.5 |
| *C. parapsilosis* 10 | 2 | 2 | 0.25 | <0.06 | 0.06 | <0.008 | 0.06 | 0.25 | 0.5 |
| *C. tropicalis* 54 | 0.06 | 0.25 | 0.06 | 0.5 | 0.015 | 0.015 | 0.06 | 0.5 | 0.5 |
| *C. tropicalis* 2 | 0.06 | 0.125 | 0.06 | 1 | 0.015 | 0.015 | 0.03 | 0.25 | 0.5 |
| *C. albicans* ATCC10231 | <0.015 | <0.008 | 0.03 | <0.06 | 0.015 | <0.008 | <0.015 | 0.5 | 0.25 |
| *C. albicans* 80 | <0.015 | <0.008 | 0.015 | 0.5 | <0.008 | <0.008 | <0.015 | <0.12 | 0.5 |
| *C. albicans* 81 | <0.015 | <0.008 | 0.03 | 0.12 | 0.03 | <0.008 | 0.03 | 0.25 | 0.25 |

*^a^*AND: anidulafungin; MF: micafungin; CAS: caspofungin; FC: 5-flucytosine; PZ: posaconazole; VOR: voriconazole; IZ: itraconazole; FZ: fluconazole; AB: amphotericin B.

**Table S2. MIC (µM) and MBEC50 (µM) values of several AMPs against *Candida* species.**

| **AMP** | ***Candida* species** | **MIC (µM)** | **MBEC_50_ (µM)** | **Reference** |
| --- | --- | --- | --- | --- |
| Human neutrophile peptide 1 & 2  (HNP1 & HNP2) | *C. albicans* | 14.5 | - | 51 |
| Human beta defensin 2  (HBD2) | several species | 1 to >58 | - | 52 |
| hLF(1-11)  [N-terminus of human lactoferrin] | several species | 9 to 36 | - | 53 |
| DS6 (artificial peptide) | *C. tropicalis* (ATCC 13803 | 11 | 45 | 54 |
| Protonectin (from the venom of the wasp *Agelaia pallipes pallipes*) | *C. albicans* | 32 | 130^a^ | 55 |
| KABT-AMP (artificial peptide) | *C. albicans* SC5314 | 11 | 22 | 56 |
| VLL-28 | several species | 12 to 50 | 50 to 100^b^ | this study |

*^a^*The reported value is not an MBEC_50_ but the concentration resulting in a significant reduction of the preformed biofilm (not quantified in the paper).

^b^ See figure 4 of this study.

**Table S3. Statistical analysis (P values) of data presented in figure 4**

|  | **VLL-28 concentration (µM)** | | | | |
| --- | --- | --- | --- | --- | --- |
|  | **6.25** | **12.5** | **25** | **50** | **100** |
| *C. albicans* ATCC10231 | 0.86 | 0.001 | < 0.001 | < 0.001 | < 0.001 |
| *C. albicans* 80 | 0.99 | 0.82 | < 0.001 | < 0.001 | < 0.001 |
| *C. albicans* 81 | 0.67 | < 0.001 | < 0.001 | < 0.001 | < 0.001 |
| *C. tropicalis* 54 | 0.76 | 0.72 | 0.004 | < 0.001 | < 0.001 |
| *C. tropicalis* 2 | 0.99 | 0.50 | 0.31 | < 0.001 | < 0.001 |
| *C. glabrata* 28 | 0.99 | 0.36 | 0.022 | < 0.001 | < 0.001 |
| *C. glabrata* 34 | 0.99 | 0.07 | 0.008 | 0.004 | < 0.001 |
| *C. parapsilosis* 3 | 0.58 | < 0.001 | < 0.001 | < 0.001 | < 0.001 |
| *C. parapsilosis* 10 | 0.77 | < 0.001 | < 0.001 | < 0.001 | < 0.001 |
| *C. krusei* 1 | 0.011 | 0.74 | 0.007 | 0.018 | < 0.001 |
| *C. krusei* 14 | 0.99 | 0.045 | 0.072 | 0.021 | 0.003 |

**Figure S1. CLSM of VLL-28 on preformed biofilm performed using a confocal microscope.** Panels a, b, c and d, e, f show biofilms of *C. albicans* and *C. parapsilosis*, respectively. Panels a and d show untreated biofilms stained with a LIVE/DEAD FungaLight Yeast Viability Kit. Panels b and e show biofilms treated with VLL-28 and stained as in a and d. Panels c and f show biofilms treated with VLL-28*.

**
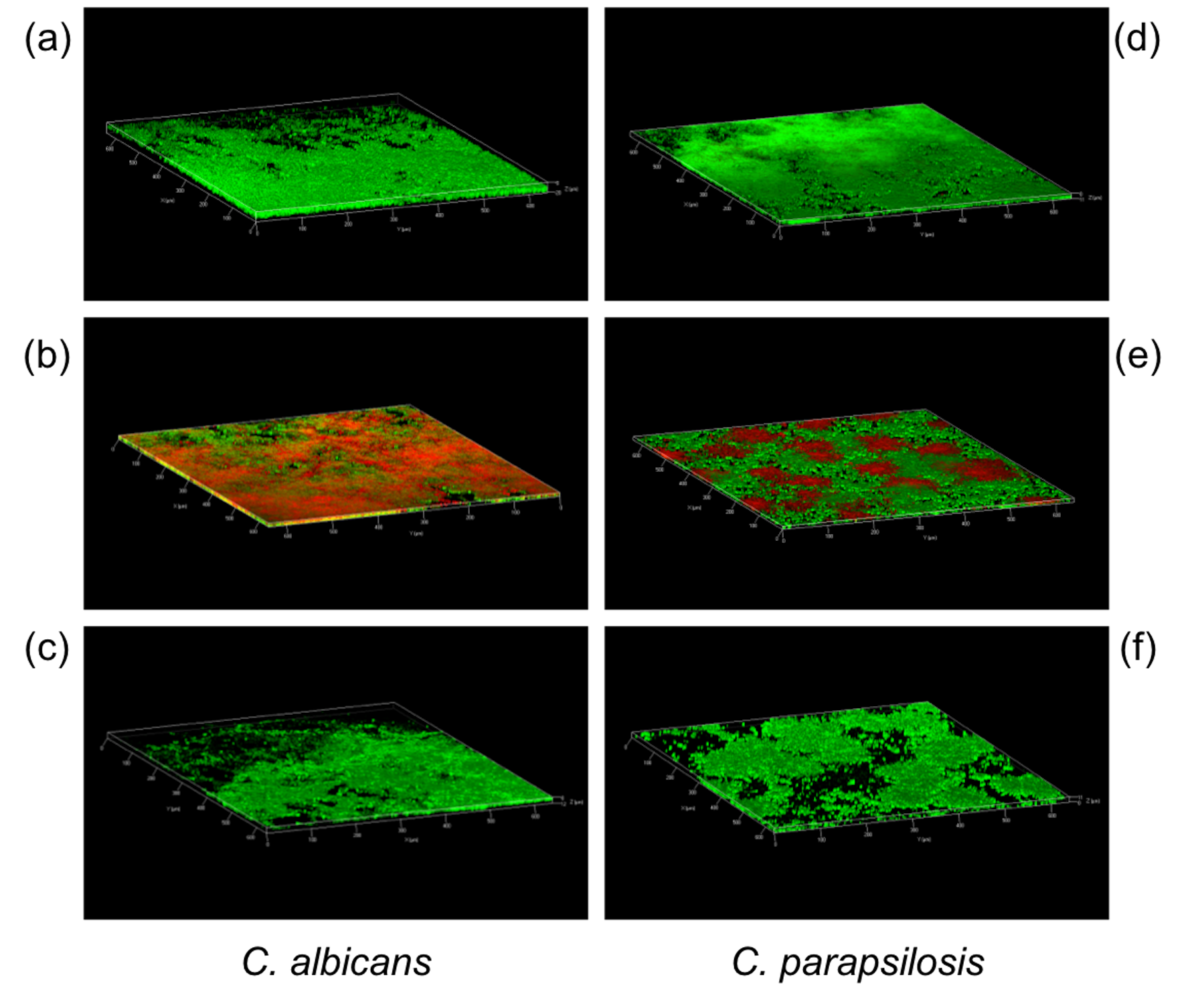
**

**Figure S2. CLSM of VLL-28 on preformed biofilm of *C. glabrata* (a and b), *C.* *tropicali*s (c and d) and *C. krusei* (e and f).** Panels a, c and e show untreated biofilms. Panels b, d and f show biofilms treated with VLL-28. All the biofilms were stained with a LIVE/DEAD FungaLight Yeast Viability Kit.


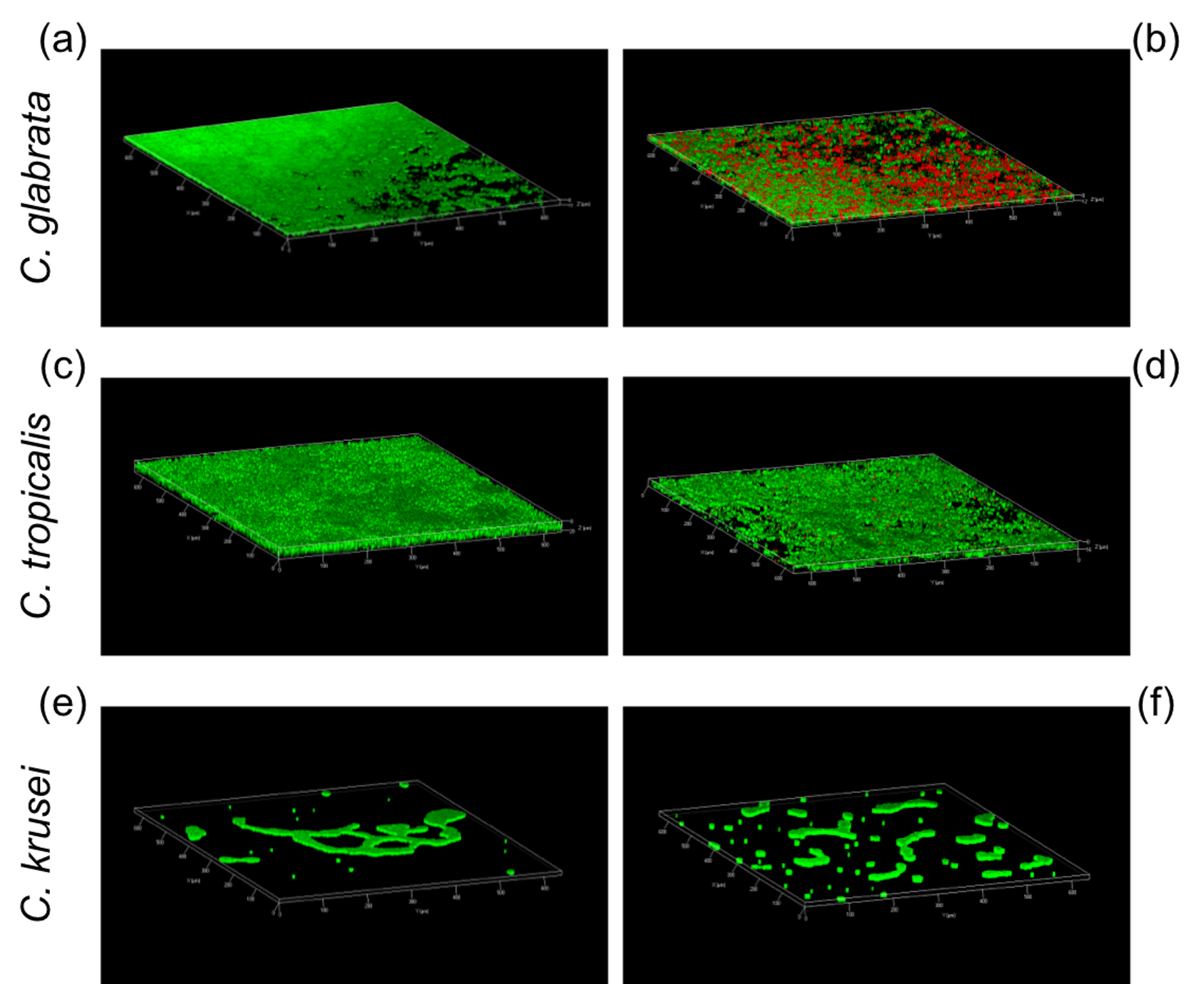

Supplement: Supplementary file 1 — Supplementary Information [file 41598_2018_35530_MOESM1_ESM.doc]
